# Role of Granulosa Cell Dysfunction in Women Infertility Associated with Polycystic Ovary Syndrome and Obesity

**DOI:** 10.3390/biom15070923

**Published:** 2025-06-24

**Authors:** Stéphanie Chauvin

**Affiliations:** Unité de Biologie Fonctionnelle et Adaptative, CNRS, Université Paris Cité, 75013 Paris, France; stephanie.chauvin@inserm.fr; Tel.: +33-157278401

**Keywords:** polycystic ovary syndrome, obesity, granulosa cells, oocyte quality, infertility

## Abstract

Infertility affects 17.5% of couples worldwide, and is notably caused in females by ovarian disorders that impact follicle development and oocyte maturation. Polycystic ovary syndrome (PCOS), affecting 8 to 13% of women of reproductive age, is a leading cause of anovulation and is characterized by arrested antral follicle development before the preovulatory stage. Reproductive issues of PCOS are often exacerbated in overweight or obese women. Obesity, which is increasingly prevalent worldwide, is also associated with anovulation, primarily due to defects in oocyte quality. Oocyte quality and competence depend on the proper activity of granulosa cells (GCs), which surround and support the oocyte. GCs produce key factors, such as 17β-estradiol, which regulate follicle growth and oocyte maturation. They also provide essential metabolic support for oocyte maturation and play a critical role in ovulation and fertilization. This review outlines the physiological role of GCs in follicle growth and maturation and explores recent advancements in understanding GCs’ molecular and physiological dysfunctions that contribute to infertility in PCOS and obesity. Improved knowledge of the endocrine mechanisms underlying follicular abnormalities in these conditions could help to predict oocyte competence and enhance assisted reproduction outcomes.

## 1. Introduction

Folliculogenesis is a highly orchestrated ovarian process that underlies female fertility. Ovarian follicles contain immature oocytes that progressively grow and acquire developmental competence, culminating in their maturation shortly before ovulation. Oocyte competence is defined by the ability to resume and complete meiosis, undergo successful fertilization, and support embryogenic development. Early stages of folliculogenesis are primarily regulated by complex signaling crosstalk between the oocyte and its surrounding somatic cells, the granulosa cells (GCs). In contrast, terminal follicular growth is governed by both pituitary-derived gonadotropins and intra-ovarian production of sex steroids. Due to their intimate and dynamic communication with the oocyte, GCs are key regulators of oocyte quality. They secrete various signaling molecules, including the sex steroid 17β-estradiol (E2), which not only supports follicular growth and oocyte maturation in an autocrine/paracrine manner but also exerts endocrine effects on tissues such as the brain, breast, and cardiovascular system [1].

Dysregulation of GC function has been implicated in several ovarian pathologies. Among them are granulosa cell tumors, which represent rare ovarian neoplasms (accounting for 2–5% of ovarian tumors) [2], and the more prevalent polycystic ovary syndrome (PCOS), which is the main cause of female infertility. PCOS affects approximately 8–13% of women of reproductive age [3] and is characterized by an accumulation of small antral follicles (2–5 mm in diameter), with premature arrest in follicular development and consequent ovulatory dysfunction [4]. Although women with PCOS often exhibit apparently normal follicular development during controlled ovarian stimulation for in vitro fertilization (IVF), dominant follicles from PCOS patients frequently yield suboptimal fertilization outcomes and impaired embryonic development [5,6]. This observation suggests that intrinsic follicular abnormalities may underlie the compromised oocyte quality observed in this population. PCOS is closely associated with metabolic dysfunction, notably obesity and insulin resistance. A significant proportion of women with PCOS are overweight or obese, with prevalence rates exceeding 50% in the United States and ranging from 30 to 60% in Europe [7]. Moreover, insulin resistance and compensatory hyperinsulinemia are common in PCOS patients, including those with a normal weight [8]. PCOS is frequently accompanied by dyslipidemia and constitutes a major risk factor for the development of type 2 diabetes and cardiovascular diseases [8].

Obesity, defined as a body mass index (BMI) ≥ 30 kg/m^2^, is a metabolic disorder whose incidence is rapidly increasing worldwide [9]. Obese women share several endocrine features with PCOS, notably female subfertility [10,11]. The prevalence of menstrual irregularity rises from 23% in women with a BMI ˂ 30 kg/m^2^ to 27% in those with a BMI > 30 kg/m^2^ [12]. In obesity, ovarian dysfunction is closely associated with reduced oocyte quality [13] and compromised endometrial receptivity, both of which may contribute to implantation failure [14,15]. Accordingly, numerous studies have demonstrated that overweight and obese women undergoing ovulation induction generate fewer oocytes and embryos and produce lower-quality blastocysts [13,16,17,18,19].

Both PCOS and obesity, which are recognized as major global public health challenges, share overlapping reproductive dysfunction that warrants greater clinical and scientific attention. Their common impact on GC function and oocyte competence suggests converging pathological mechanisms within the ovarian follicular environment. This review aims to provide a comprehensive and up-to-date overview of the pivotal role of GCs in regulating follicular development and oocyte maturation. We will also summarize current molecular insights into how GC dysfunction within the follicles of women with PCOS or obesity contributes to impaired fertility outcomes. A deeper understanding of these mechanisms may ultimately lead to the development of safer and more targeted therapeutic strategies to improve reproductive success in affected women.

## 2. Functional Importance of Granulosa Cells in Follicular Growth and Oocyte Maturation

Oocyte development and maturation involve a series of highly coordinated processes, primarily orchestrated by GCs, particularly cumulus granulosa cells (CGCs). These processes are essential to ensure the acquisition of oocyte competence. In primordial follicles, oocytes are arrested at the diplotene stage of meiosis I and considered meiotically incompetent [20]. Upon recruitment into the growing follicular pool, the oocyte undergoes rapid growth, with its diameter increasing from approximately 40 µm in human primary follicles to approximately 100 µm in antral follicles, and gradually reaching approximately 140 µm in preovulatory follicles [21]. Oocyte maturation encompasses both nuclear and cytoplasmic changes [22]. Nuclear maturation includes chromosomal segregation, followed by progression through meiosis I and arrest at metaphase II until fertilization [23]. Cytoplasmic maturation supports fertilization and embryo development by facilitating the accumulation of maternal mRNAs, proteins, and nutrients and the rearrangement of organelles [24]. The number of mitochondria, the main site of adenosine triphosphate (ATP) generation, increases dramatically, with approximately 200 in early stages to approximately 400,000 mitochondrial DNA (mtDNA) copies in mature oocytes [25]. Moreover, mitochondria become structurally organized [26], forming functional complexes with the endoplasmic reticulum to regulate calcium homeostasis, a process critical for effective embryo development [27]. The Golgi apparatus expands during maturation and produces vesicles giving rise to cortical granules that prevent polyspermy by modifying the oocyte’s extracellular matrix (ECM) structure after fertilization [23]. Altogether, these nuclear and cytoplasmic changes are vital for achieving full oocyte maturity and ensuring the successful formation of developmentally competent blastocysts [28].

### 2.1. Oocyte Maturation Depends on Proper Oocyte–GCs Crosstalk

Oocyte maturation relies on two primary modes of communication between the oocyte and GCs, especially CGCs. The first mechanism consists of direct cell–cell contact via transzonal projections (TZPs), which are cytoplasmic extensions from CGCs that cross the zona pellucida to establish contact with the oocyte plasma membrane [29]. In mammals, including humans, the tips of TZPs form gap junctions that enable the bidirectional exchange of small molecules such as ions, cyclic nucleotides, metabolites, amino acids, and even RNA transcripts [30,31,32,33]. This molecular exchange is essential for supporting oocyte growth and maintaining meiotic arrest [34]. The second mechanism involves paracrine signaling, whereby one cell type secretes cytokines or growth factors that bind to specific receptors on the other, initiating intracellular signaling cascades [35]. Temporal single-cell RNA sequencing (scRNA-seq) analyses of human oocytes and GCs across follicular development have revealed stage-specific gene expression patterns, highlighting the reciprocal nature of oocyte–GC communication [36]. For example, neurotrophin 4, which promotes follicle-stimulating hormone (FSH) receptor (FSHR) expression in mouse GCs [37], is enriched in human antral-stage oocytes and this coincides with elevated FSHR expression in GCs from corresponding follicles. The concomitant expression of ligands and their cognate receptors between the oocytes and GCs underscores a bidirectional regulation network in which oocyte-derived factors modulate GC function, which, in turn, influence oocyte development [36]. Moreover, this study showed that during the transition from primordial to primary follicles, in addition to the well-characterized mammalian target of rapamycin (mTOR)–phosphatidylinositol 3-kinase (PI3K) signaling pathways, both oocytes and GCs activate additional signaling pathways, including those mediated by insulin, gonadotropin-releasing hormone (GnRH), and neurotrophins [36].

### 2.2. Oocyte Orchestrates Granulosa Cell Functions

Throughout folliculogenesis, the oocyte plays a central regulatory role in the growth, differentiation, and survival of GCs. During the early preantral stage, the oocyte stimulates GCs to express the kit ligand, which promotes oocyte growth via its receptor c-Kit. Once the oocyte reaches a critical size, it down-regulates kit ligand expression to prevent further expansion [36,]. At the antral stage, the oocyte contributes to the formation of the follicular antrum, a cavity filled with secretions from both the oocyte and GCs, which serves as a nutritive and protective microenvironment [38]. Evidence from oocytectomized cumulus–oocyte complexes (COCs) cultures, as well as co-cultures of fully-grown denuded oocytes and GCs from bovine, porcine, or ovine follicles, have demonstrated that oocyte-secreted factors regulate several critical aspects of GC functions. By their close proximity, CGCs are particularly responsive to oocyte-secreted factors. These factors promote GC proliferation [39] and suppress steroidogenic activity [34,40,41]. They also regulate CGC differentiation by preventing their premature luteinization and transition into mural GCs (MGCs) [42], stabilize ECM by protecting it against proteolytic degradation [35], and support mucification [25,36] through the production of prostaglandins [33,34]. In addition, in vitro studies on porcine COCs have shown that oocyte-secreted factors modulate androgen action by down-regulating androgen receptor expression in MGCs, thereby reducing both DNA synthesis and progesterone production [43].

Among the most well-characterized oocyte-derived factors are growth differentiation factor-9 (GDF9) and bone morphogenetic protein-15 (BMP15), both members of the TGFβ-superfamily. Their physiological significance has been demonstrated in animal models with reproductive defects: in sheep or mice, a genetic deficiency of Bmp15 or Gdf9, respectively, prevents folliculogenesis from progressing beyond the primary stage [35]. Similarly, mutations in human GDF9 and BMP15 genes are associated with premature ovarian insufficiency [42]. Studies in rat CGCs have shown that GDF9 and BMP15 induce the expression of the natriuretic peptide precursor type C (NPPC) receptor, Npr2 [44], as well as inosine monophosphate dehydrogenase, both of which contribute to elevated cyclic guanosine monophosphate (cGMP) levels in CGCs and thus the maintenance of oocyte meiotic arrest [32]. Numerous studies have further identified GDF9 and BMP15 as key regulators of GC proliferation and differentiation [33,41]. Transcriptomic analyses of CGCs from BMP15- and GDF9-mutant mice, along with in vitro treatments of intact bovine COCs with recombinant forms of these proteins, have shown that they regulate, mostly in a coordinated manner, gene networks involved in CGC metabolism including glycolysis, sterol biosynthesis, inositol metabolism, and the pentose phosphate pathway [41,45]. They also activate alternative signaling pathways that support CGC growth and survival [46]. GDF9 and BMP15 have been shown in many species, including human, to inhibit FSH-induced luteinizing-hormone (LH) receptor mRNA expression in MGCs [39], preventing their luteinization.

### 2.3. Pivotal Role of Granulosa Cells in Oocyte Quality

**Figure 1:**
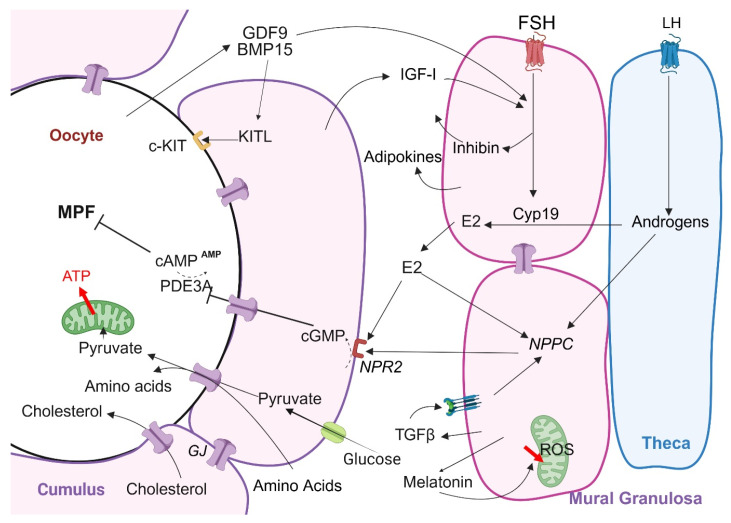
Reciprocal interactions between oocyte and granulosa cells regulating follicular development and meiotic maturation. Within the antral follicle, mural and cumulus GCs communicate with the oocyte through gap junctions (GJs). The oocyte releases paracrine factors (growth differentiation factor-9, GDF9, and bone morphogenetic protein-15, BMP15) that regulate GC proliferation and differentiation. These factors, along with insulin-growth factor I (IGF-I) and follicle-stimulating hormone (FSH), promote GC growth and steroidogenesis. FSH also activates key signaling pathways and increases estradiol (E2) production. E2, in turn, influences gene expression and signaling pathways that maintain meiotic arrest in the oocyte. GCs supply essential nutrients and protective molecules (e.g., TGFβ, melatonin) to support oocyte health and maturation. Most information is from rodents. LH, luteinizing hormone; NPPC, natriuretic peptide precursor and NPR2, its receptor; cGMP, cyclic guanosine monophosphate; cAMP, cyclic adenosine monophosphate; MPF, maturation-promoting factor; ATP, adenosine triphosphate; TGFβ, transforming growth factor β; PDE3A: phosphodiesterase 3A; ROS, reactive oxygen stress; KITL, kit ligand and its receptor, c-KIT. Created in BioRender. Le ciclé, C. (2025) https://BioRender.com/s2tr84f. Student Plan-Academic; 6 June 2025.

GCs play a pivotal role in maintaining oocyte quality by regulating meiotic arrest, providing metabolic support, and protecting against oxidative stress (Figure 1). Their functions are crucial for oocyte competence and subsequent embryonic development.

#### 2.3.1. Maintenance of Meiotic Arrest and Regulation of COC Expansion

GCs contribute to the maintenance and regulation of meiotic progression through the NPPC/NPR2 signaling pathway [47]. Studies on mouse and human follicles have shown that MGCs produce NPPC, which binds to NPR2 receptors on cumulus cells, leading to the production of cGMP. This cGMP diffuses to the oocyte through gap junctions, where it inhibits phosphodiesterase 3A (PDE3A), thereby maintaining elevated levels of cyclic adenosine monophosphate (cAMP), a critical factor for sustaining meiotic arrest [48,49]. Genetic disruption of either the *Npr2* or *Nppc* gene in mice leads to premature meiotic resumption of oocytes [44], underlining the key role of the NPPC/NPR2 system for maintaining oocyte meiotic arrest [32].

Upon FSH stimulation, GCs predominantly secrete E2, which reinforces meiotic arrest in oocytes by up-regulating NPPC and NPR2 expression in GCs and regulating several meiotic gene transcriptions in oocytes [50]. MGCs express TGFβ members (TGFβ 1/3) and signaling entities (Smad3), which further promote NPPC expression, inhibiting meiotic resumption [51]. GCs also secrete factors like the kit ligand [33,40]), activin, and inhibin (in response to oocyte signals [44,52]), supporting oocyte growth and maturation [53]. At the LH surge, estrogen and androgen receptors are down-regulated in MGCs [54,55,56], reducing NPPC production. LH also inactivates NPR2 in CGCs [57] via epidermal growth factor (EGF) receptor (EGFR) signaling and elevated intracellular calcium (Ca2+i), leading to reduced cGMP, PDE3A activation, and meiotic resumption. LH-mediated extracellular signal-regulated kinase (ERK) pathway activation inhibits connexin 43 translation [49,58], thereby disrupting TZP gap junctions and further reducing the oocyte’s cGMP [45,49,59]. In addition, CGCs secrete endothelin-1, enhancing ERK signaling to promote meiosis in preovulatory mouse oocytes [60]. It has been shown in mouse COCs that FSH, GDF9, and BMP15 induce the expression of EGFR [61], and that LH further triggers EGF-like ligands (e.g., amphiregulin [49,62]), promoting COC expansion [62] and meiotic progression [31]. EGFR signaling up-regulates genes involved in ECM formation (e.g., hyaluronic acid synthase 2, cyclooxygenase 2, and gremlin [), the expression of which is correlated with embryo quality [63].

#### 2.3.2. A Metabolic Support for Oocyte Maturation

The absence of surrounding cumulus cells during in vitro maturation, particularly during the initial phase, leads to metabolic dysregulation in oocytes and compromises their developmental competence, as evidenced by reduced fertilization rates and impaired embryonic development in multiple mammalian species, including bovine, porcine, and murine models [64]. Oocyte has a low capacity for glucose metabolism, due to the lack of expression of the glucose transporter GLUT4, and the low activity of phosphofructokinase, a rate-limiting glycolysis enzyme, together with the presence of naive (roundish-oval like structures with unstructured cristae) mitochondria [25,65,66]. By contrast, CGCs exhibit high glycolytic activity, converting glucose into pyruvate (mainly via the anaerobic glycolytic pathway) which is then transferred to the oocyte. This pyruvate supports, through oxidative phosphorylation, the production of ATP, which is critical for oocyte meiotic resumption and developmental potential [67]. The metabolomic profiling of mouse oocytes undergoing in vivo maturation revealed a significant increase in amino acids, carbohydrates, and nucleotides during meiotic maturation, while lipid metabolites exhibited a pronounced decrease [67]. Studies in mice have demonstrated that CGCs actively uptake amino acids and cholesterol (or synthesize it from acetate) and subsequently transfer these metabolites to the oocyte [41]. Additionally, CGCs are capable of producing ATP via fatty acid oxidation, a more efficient ATP source than glycolysis [31]. CGCs provide the oocyte with nutrients to facilitate the progression of maturation, notably nuclear maturation [41,68].

#### 2.3.3. Protection from Oxidative Stress

Anatomically, CGCs form a critical biological barrier between the oocyte and its environment, including the follicular fluid content, which contains various components such as fatty acids. In vitro studies on bovine oocytes have demonstrated that CGCs protect oocytes from the detrimental effects of long chain saturated-fatty acids, which negatively impact oocyte competence [69,70]. Given the limited intrinsic antioxidant capacity of oocytes, CGCs contribute significantly to oocyte redox homeostasis, protecting it from oxidative stress, an important factor in oocyte quality. Both MGCs and CGCs synthetize melatonin which confers protection to human oocytes by preserving mitochondrial membrane potential [71], enhancing antioxidant enzyme activities, and directly scavenging free radicals [72,73]. The protective role of melatonin is further supported by evidence showing a positive correlation between elevated melatonin levels in follicular fluid and oocyte quality [74]. Moreover, CGCs play a vital role in regulating the oocyte’s intracellular pH (pHi) under acidic conditions, maintaining pHi homeostasis until the oocyte acquires the capacity for autonomous pHi regulation [75].

## 3. PCOS and Obesity: Two Distinct Metabolic Diseases Sharing Infertility Issues

Genetic predisposition [76], environmental impact, or a combination of these is considered to play a critical role in the pathophysiology of PCOS, particularly by promoting excessive androgen synthesis. Significant ethnic variations have been reported in PCOS clinical manifestations, which may be influenced by differences in nutritional and lifestyle habits [77]. In vitro studies revealed that thecal cells isolated from polycystic ovaries produce up to 20-fold-higher levels of androgens, compared to normal ovaries, primarily due to the increased expression of key steroidogenic enzymes. Women with PCOS exhibit an altered gonadotropin profile, characterized by an elevated LH/FSH ratio, generally exceeding 1 [12,78]. This hormonal imbalance results from impaired negative feedback at the level of the hypothalamic–pituitary unit leading to an increased frequency and amplitude of GnRH pulses, and subsequent LH secretion [79]. According to the Rotterdam criteria, a diagnosis of PCOS requires the presence of at least two of the following features: oligo- or anovulation, clinical/biological hyperandrogenism, and polycystic ovarian morphology on ultrasonography [80]. PCOS is diagnosed after excluding other causes of hyperandrogenism, such as adult-onset congenital adrenal hyperplasia, hyperprolactinemia, and androgen-secreting neoplasms [80]. Based on these criteria, four distinct PCOS phenotypes have been defined: Phenotype A (hyperandrogenism, ovulatory dysfunction, and polycystic ovary (PCO) morphology); Phenotype B (hyperandrogenism and ovulatory dysfunction); Phenotype C (hyperandrogenism and PCO morphology); and Phenotype D (ovulatory dysfunction and PCO morphology) [81,82]. Hence, not all women with PCOS exhibit hyperandrogenism. The classic phenotypes (A and B) are associated with a more severe metabolic profile, including a higher prevalence of metabolic syndrome and elevated BMI, compared to normo-androgenic phenotypes. The PCOS phenotype distribution varies depending on geographic region or genetic background. In France and Mexico, Phenotypes A and B account for approximately 61–70% of women with PCOS [82,83], whereas in Denmark, 72% of women with PCOS exhibit Phenotype C. Phenotype D (non-hyperandrogenic PCOS) affects between 10% and 32% of women, depending on the region of the world [82]. PCOS is now widely recognized as a metabolic disorder. Regardless of BMI, many affected individuals develop insulin resistance and compensatory hyperinsulinemia, which affects approximately 60–70% of women with PCOS, while the overall prevalence is only 10–25% [84,85]. Elevated androgen concentrations contribute to visceral adiposity by disrupting the balance between lipolysis and lipogenesis in adipose tissue, leading to an increased release of free fatty acids and inflammatory cytokines [86]. Androgens also stimulate the growth of preantral follicles and promote excessive follicular recruitment [87]. However, the progression of these follicles to the antral stage is impaired due to insufficient FSH levels and androgen-mediated inhibition of FSH-induced aromatase activity in larger follicles [88]. This leads to an accumulation of early antral follicles, which in turn elevates anti-Müllerian hormone (AMH) levels. AMH is exclusively produced by GCs up to the small antral stage. This hormone is depicted to impede FSH-induced GC growth and steroidogenesis [89] and to promote inhibin synthesis that reduces FSH secretion [90]. AMH also contributes to PCOS pathophysiology by acting centrally to enhance GnRH and LH pulsatility and locally by down-regulating FSH receptor expression in GCs [91]. Insulin further exacerbates this hormonal imbalance. It acts at the hypothalamic level to stimulate GnRH secretion and at the hepatic level to reduce sex hormone binding globulin (SHBG) synthesis, thereby increasing the bioavailability of circulating androgens [86]. Within the ovary, insulin cooperates with LH to stimulate androgen synthesis and represses the synthesis of insulin-like growth factor (IGF) binding protein-1 in GCs, increasing the free IGF-1 concentration. This local increase in IGF-1 cooperates with insulin to enhance proliferation and steroidogenesis of thecal and granulosa cells [79]. Additionally, an up-regulation of IGF-1 receptors has been observed in GCs from insulin-resistant PCOS patients [92], further enhancing ovarian insulin activities. Collectively, these alterations impair follicular development by arresting growth at the small antral stage.

On the other hand, obesity is characterized by excessive lipid accumulation and elevated circulating leptin levels, both of which contribute to insulin resistance and compensatory hyperinsulinemia, leading to systemic inflammation and oxidative stress [12,93]. Obese women have a higher incidence of menstrual irregularities and anovulatory infertility compared to normal-weight women. The relative risk of anovulation starts increasing at a BMI of 24 kg/m^2^ and continues to rise with increasing BMI [93,94]. Obesity impairs fertility through multiple mechanisms, including altered oocyte development, reduced fertilization rates, compromised embryo quality, and impaired implantation [95]. At the neuroendocrine level, obesity disrupts the hypothalamic–pituitary–ovarian axis. Indeed, the increased aromatization of androgens in adipose tissue elevates circulating estrogen concentrations, which in turn suppress GnRH secretion by negative feedback, leading to irregular or anovulatory cycles [95]. Obesity attenuates the LH pulse’s amplitude rather than its frequency [12]. Experimental studies have demonstrated that the combined elevation of circulating insulin and fatty acids reduces basal GnRH-stimulated LH concentrations in lean eumenorrheic women [96,97]. Moreover, the enhanced metabolic clearance of endogenous LH contributes to reduced LH levels in women with obesity [12]. Additionally, elevated leptin concentrations in obesity may induce leptin resistance in the hypothalamus, further impairing GnRH and subsequent LH secretion [12]. Androgen excess is also observed in obese women without PCOS. A cross-sectional study of 199 premenopausal women with severe obesity reported hyperandrogenemia in 32% of women without PCOS and 45% of those with PCOS [98]. This increase in androgen levels is partly attributed to the enhanced conversion of androstenedione to testosterone in subcutaneous adipose tissue, a process that correlates positively with BMI [99]. In lean women, supraphysiological levels of testosterone reduce LH secretion, contrary to women with PCOS who display a higher threshold for androgen-induced inhibition of GnRH–LH because of chronic exposure to elevated androgen levels [12].

Obesity exacerbates PCOS’s clinical features, including menstrual disturbances, dysfunctional uterine bleeding, and hirsutism [7,100]. Clinical insulin resistance occurs in approximately 70–80% of obese women with PCOS [82]. However, even in the absence of overt metabolic symptoms, obese women with PCOS exhibit disruptions in glucose homeostasis at the follicular level, particularly within CGCs [101]. Obesity does not invariably exacerbate insulin resistance in PCOS [102]. The insulin resistance prevalence among lean women with PCOS widely varies depending on the geographic region and genetic background, from 38.3% to 83.3% in North America, France, Turkey, Australia, and India [103,104,105,106,107,108]. In contrast, lean women with PCOS in Sweden and Denmark do not develop insulin resistance [109,110]. A study on lean women with PCOS showed hyperleptinemia and reduced lipolytic sensitivity, which preserves subcutaneous adipose tissue [111]. Importantly, increased inflammation markers are observed in both lean and obese PCOS patients compared to BMI-matched controls, indicating that low-grade inflammation is more closely related to PCOS pathology itself rather than obesity [112]. Moreover, obesity appears not to be a primary cause of PCOS, contrary to some common ideas, since a significant proportion of women with PCOS maintain a normal weight [113]. A recent meta-analysis of 58 studies found only a modest association between obesity and PCOS, estimating a 0.4% increase in PCOS prevalence for every 1% rise in obesity prevalence [114]. These findings support the presence of intrinsic etiological factors underlying PCOS, independently of obesity. In contrast, insulin resistance is a common feature between (lean and obese) PCOS and obese women and may exacerbate PCOS-related metabolic and reproductive abnormalities when PCOS and obesity coexist.

## 4. Granulosa Cell Dysfunction Is Associated with Subfertility in Obese Women with or Without PCOS

### 4.1. GC Dysfunction in Obese Women

Oocyte quality is commonly evaluated based on the ability of the oocyte to form an embryo capable of successful implantation [115]. A substantial body of evidence indicates that obesity has a detrimental impact on oocyte quality [13]. Among studies on obese women, few investigations specifically classified patients by age, a critical determinant of embryo competence [16]. Metwally and colleagues demonstrated a significant association between obesity and reduced embryo quality in women under 35 years of age, with younger obese women showing a lower chance of cryopreserved embryos and requiring higher dose of gonadotropins for ovarian stimulation [16]. In contrast, Bellver and colleagues reported impaired IVF outcomes in obese women of the same age range, without a corresponding decrease in embryo quality [116]. Nonetheless, they similarly observed that obese women undergoing ovarian stimulation programs required higher gonadotropin doses and longer treatment durations [116].

Obesity induces alterations in the follicular environment that may compromise oocyte quality. Similar to changes observed in circulation, increased BMI is associated with modifications in follicular fluid composition, including elevated concentrations of insulin, C-reactive protein [17], leptin [12,117], interleukin-6, and tumor necrosis factor-α [15], relative to normal-weight individuals. Such alterations have been linked to reduced oocyte developmental competence [17]. Obese women also exhibit increased levels of adipokines, such as leptin, in the follicular fluid [118], which are often correlated with heightened ROS levels and oxidative stress [119,120]. At physiological concentrations, leptin plays a role in promoting steroidogenesis and follicular maturation; however, at supraphysiological levels, it may exert inhibitory effects [121]. Thus, elevated leptin, produced predominantly by adipocytes and locally by GCs, could adversely affect folliculogenesis and oocytes. Indeed, free fatty acids, susceptible to peroxidation, may generate ROS that induce apoptosis by disrupting the mitochondrial and endoplasmic reticulum integrity in both GCs and oocytes [45] and promote suboptimal embryonic development [122]. High concentrations of lipids and pro-inflammatory cytokines have been shown to impair oocyte maturation, as demonstrated by compromised metaphase II progression in mouse oocytes exposed to lipid-rich follicular fluid in vitro [123]. Consistent with these findings, studies on in vitro maturated human oocyte revealed that oocytes from obese patient exhibit spindle defects and aberrant chromosome segregation [124].

Alterations in the follicular fluid composition may stem from intrinsic GC dysfunction. A high-throughput proteomic analysis revealed that GCs from obese follicles display mitochondrial dysfunction, marked by enhanced electron transfer and oxidoreductase activities, both indicative of metabolic dysregulation [117]. In obese mouse models, COCs have been shown to exhibit normal mtDNA content but reduced mitochondrial membrane potential and elevated autophagy levels, compared to lean mice [125]. Additionally, transcriptomic analyses comparing GCs from obese and normal-weight women revealed down-regulation of the FSH receptor in the obese group [126], consistent with clinical observations of increased FSH requirements for effective ovarian stimulation in obese patients undergoing IVF [13,17]. Furthermore, in vitro studies using high-fat-diet-induced obese mice reported increased GC apoptosis [127] and impaired activation of the AKT/FOXO3a signaling pathway [128], further increasing GC abnormalities in the ovaries of obese women.

Nonetheless, only a few studies have investigated ovarian abnormalities specifically in obese women. Most existing research has focused on systemic metabolic comorbidities of obesity in patients or alterations in hypothalamic circuitries in animal models [129]. There is a clear need for studies exploring the direct impact of obesity on GC function, follicular development, and oocyte quality in humans.

### 4.2. GC Dysfunction in Lean or Obese Women with PCOS

#### 4.2.1. Elevated Oxidative Stress and Apoptosis

At the follicular level, GCs from lean or obese women with PCOS display functional abnormalities. One consistent finding is an aberrant increased LH sensitivity in GCs, which leads to premature follicular differentiation [92]. In obese women with PCOS, GCs also exhibit reduced FSH receptor expression, a defect consistent with the clinical requirement of higher total doses and/or prolonged administration of FSH during controlled ovarian IVF protocols [126], as observed for obese patients [13,17]. Furthermore, GCs from lean and overweight women with PCOS undergoing IVF treatments have been described to display increased levels of apoptotic markers such as Forkhead box O3 (FOXO3), Annexin V, and p53, alongside elevated concentrations of ROS [5,130,131,132,133]. In murine models, oxidative stress was shown to activate the c-Jun N-terminal Kinase (JNK) signaling pathway, which enhances FOXO1 activity and promotes GC apoptosis [134]. Interestingly, treatment with growth hormone during controlled ovarian stimulation in lean women with PCOS (with hyperandrogenism and insulin resistance) was associated with a reduction in ROS levels back to the levels observed in non-PCOS patients [133]. This effect was reported to be mediated through the restoration of PI3K/AKT signaling, which plays a critical role in GC survival [133]. Mitochondrial dysfunction has also been implicated in PCOS-related GC pathology. Studies involving rodent PCOS models as well as human GCs obtained after IVF from lean or overweight women with PCOS revealed structural mitochondrial abnormalities and significantly reduced mtDNA copy numbers [135,136]. These alterations are accompanied by impaired ATP production and increased mitochondrial ROS generation [5,135,137]. ROS have been shown to trigger signaling cascades, including the Nuclear factor erythroid 2 related factor-2 (Nrf2)/heme oxygenase-1 (HO-1) pathway, leading to a down-regulation of antioxidant expression such as that of superoxide dismutase, catalase, and HO-1. More recently, ROS have also been implicated in the activation of the p21-activated kinase/β-catenin pathway [138].

The combination of elevated ROS levels and reduced ATP content in GCs negatively impacts follicular competence. Specifically, studies have reported a significant inverse correlation between oxidative stress markers in GCs and the ratio of high-quality embryos to retrieved oocytes in lean or overweight women with PCOS undergoing IVF [6,137].

#### 4.2.2. Metabolic Dysfunction

GCs isolated from both mature and immature follicles of overweight PCOS patients also demonstrated reduced energy production compared to those from control women [6]. Glycolysis function is altered in GCs from both lean and overweight women with PCOS, as evidenced by reduced levels of pyruvate and lactate in their follicular fluid [78], alongside a down-regulation of genes encoding glycolysis-related enzymes such as glucose transporter type 1 or lactate dehydrogenase A [5,6,136]. Additionally, GCs from lean and overweight women with PCOS have been described to exhibit dysfunctional mitochondria with abnormal morphology [78,136]. A marked decreased in mitochondrial respiration ability is accompanied by a down-regulation of Sirtuin 3 (a key regulator of mitochondrial metabolism and homeostasis [139]) expression, which may be correlated with unsuccessful pregnancy outcomes following IVF and embryo transfer in these women [136]. In vitro studies using a tumoral granulosa cell line (KGN) have highlighted the functional importance of the deacetylase Sirtuin 3, by showing that its deficiency disrupts mitochondrial respiration and promotes ROS generation [136]. Furthermore, quantitative acetyl-proteomics analysis of GCs from lean women with PCOS revealed widespread alterations in the lysine acetylation of proteins involved in key metabolic pathways, including glycolysis and the tricarboxylic acid cycle [140], potentially impairing enzyme activity and metabolic homeostasis.

Genes involved in lipid synthesis and oxidation, such as SREBP1 or ACC1, were found to be up-regulated in GCs from women with PCOS and insulin resistance, as opposed to non-insulin-resistant women with PCOS, potentially leading to excessive ROS production [92]. Both serum and follicular fluid from lean and obese women with PCOS showed reduced levels of free carnitine [141]. Carnitine is a critical component of fatty acid metabolism that facilitates fatty acid transport to mitochondria for β-oxidation and ATP production [142]. Accordingly, supplementation with L-carnitine in follicle culture media has been shown to enhance lipid utilization, increase energy availability, and improve oocyte and embryo developmental competence [142].

The majority of women with PCOS exhibit peripheral insulin resistance. At the ovarian level, GCs from both lean and overweight/obese women with PCOS have been shown to display a selective insulin resistance, characterized by impaired metabolic responses while preserving insulin-stimulated steroidogenesis and mitotic activity [143,144,145]. Molecular analyses have revealed that this resistance is associated, depending on the PCOS cohort, with the down-regulation of the insulin receptor β-subunit in lean women with PCOS, along with defects in post-receptor signaling [85,92,107]. Transcriptomic profiling of CGCs from overweight and obese women with PCOS has demonstrated significant alterations in genes involved in metabolic processes [146,147]. In lean women with PCOS, 487 genes were differently expressed compared to lean controls, notably showing a reduced expression of transcription factors from the Wnt/β-catenin and MAPK signaling pathways, which are key regulators of cell proliferation, differentiation, and fate determination [148]. In obese women with PCOS, 174 genes were differently expressed relative to obese non-PCOS controls, including an up-regulation of insulin and leptin receptor genes and down-regulation of genes involved in mitochondrial metabolism [148]. Furthermore, a meta-analysis of transcriptomic data across multiples tissues revealed that lean and overweight/obese women with PCOS exhibit largely distinct signaling pathway profiles, with only 6.7% shared enriched pathways, highlighting the molecular heterogeneity of women with PCOS depending on whether they are obese or not [149].

Given the essential role of GCs in providing metabolic support and antioxidant protection to the oocyte, dysregulated glucose and lipid metabolism and elevated ROS levels result in a deficient energy supply for both oocytes and GCs and increased GC apoptosis. This dysfunction directly impairs oocyte quality and contributes to reduced IVF success rates in women with PCOS.

#### 4.2.3. Contribution of the Oocyte Inflammatory Environment

PCOS is associated with a low-grade systemic pro-inflammatory state that also affects the ovarian follicular environment. Transcriptomic analysis of GCs from lean and obese women with PCOS has shown an up-regulation of inflammation-related genes compared to lean controls [146], with more pronounced expression in the obese subgroup [150]. Furthermore, in vitro exposure of GCs from lean women to dihydrotestosterone (DHT) significantly increased the expression of pro-inflammatory cytokines, such as IL6 and CCL20 [150], indicating the contribution of hyperandrogenism to ovarian inflammation.

Although cytokines play critical roles in normal ovulation and corpus luteum formation, their excessive presence within the follicular environment may lead to an overexpression of proangiogenic genes in GCs. This could disrupt COC expansion and compromise oocyte competence. Proteomic analyses of follicular fluid from obese women with PCOS revealed the overexpression of proteins involved in inflammatory pathways [147]. Elevated levels of cytokines, such as interleukins and tumor necrosis factor alpha, have been consistently detected in both GCs and matched follicular fluid from lean and obese women with PCOS [150,151]. These inflammatory mediators contribute to the negative follicular environment, aggravating oxidative stress and cellular dysfunction, and further exacerbate oocyte dysfunction.

#### 4.2.4. Oocyte–GC Communication Defects

In women with PCOS, communication between the oocyte and GCs is altered, contributing to abnormal follicular development [152]. PI3K signaling plays a central role in this dysregulation. Specifically, the expression of aquaporin-9, a water channel protein essential for follicular fluid accumulation during antrum formation in mice [153], is significantly reduced in GCs from women with PCOS compared to infertile women with tubal factor infertility [154]. In vitro studies have shown that the treatment of GCs from the preovulatory follicles of lean women with PCOS with DHT leads to the down-regulation of aquaporin 9 expression via the PI3K pathway [154], suggesting that hyperandrogenism in PCOS further impairs aquaporin function, thereby compromising follicular maturation. scRNA-seq has provided deeper insights into the molecular defects affecting oocyte–GC communication. Analysis of COCs from women with PCOS revealed significant transcriptomic alterations in CGCs at various stages of oocyte maturation [155]. Notably, at the germinal vesicle stage, CGCs from PCOS follicles showed the dysregulated expression of genes involved in cell proliferation, gap junction communication, and oxidative stress responses [155], key processes essential for proper oocyte development and competence. In overweight women with PCOS, follicular fluid also displays dysregulated expression of proteins involved in ECM remodeling, vascular development, and oxidative stress [156], which likely impairs COC expansion.

#### 4.2.5. Alterations in GC-Secreted Factors

GC dysfunction in PCOS can also be assessed indirectly by analyzing the concentrations of GC-secreted factors in follicular fluid. In both lean and obese women with PCOS, follicular fluid is characterized by unchanged [157] or lower levels of E2 [126,158,159] and lower levels of progesterone (P4) [157,158,160,161] and melatonin [158], while the levels of testosterone (T) [157,158,159,160,161] and AMH [162] are markedly increased. These imbalances reflect disrupted steroidogenesis, paracrine signaling, and antioxidant support within the follicular environment. Several studies have identified a positive correlation between the E2/T ratio in follicular fluid and live birth rates following IVF, underscoring the importance of this hormonal balance as a predictive marker of reproductive outcomes [163]. Excessive T levels result from the increased stimulation of thecal cells by LH and insulin [164]. Furthermore, heightened androgen output from thecal cells combined with increased androgen receptor expression in GCs leads to enhanced AMH production in PCOS patients [165,166]. This, along with an excess of growing follicles [161,162,167], contributes to the elevated AMH concentrations observed in PCOS. The altered expression of key steroidogenic enzymes in GCs partly accounts for the reduced P4 and E2 levels in the follicular fluid of overweight and obese women with PCOS [126,159,161]. This impairment may be exacerbated by the intriguing inability of E2 to regulate its canonical genes in the GCs of overweight women with PCOS [161]. Collectively, the combination of a low E2/T ratio and high AMH levels may impair dominant follicle selection and disrupt normal folliculogenesis [168]. In addition, the reported deficiency in melatonin further compromises the oocyte developmental potential of PCOS follicles [158]. Leptin levels are also significantly elevated in both the serum and follicular fluid of women with PCOS compared to controls, independently of BMI, potentially having a detrimental impact on oocyte maturation [169]. Additionally, hyperandrogenism directly affects oocyte maturation. Excessive T and androgen receptor expression in GCs from women with PCOS have been associated with reduced in vitro maturation, lower fertilization rates, and impaired embryo development [31]. These oocytes, although morphologically mature (metaphase II) exhibit altered expression of genes critical for meiotic progression [170]. This suggests that intrinsic abnormalities in PCOS oocytes may persist through ovulation, even when follicular development is pharmacologically induced. This is further supported by evidence from the dehydroepiandrosterone-induced PCOS-like mouse model, which demonstrated up-regulation of the NPPC/NPR2 signaling system via direct androgen receptor binding to their gene promoters [171].

## 5. How to Improve the Current Therapeutic Strategies

### 5.1. Current Therapeutic Approaches

Obesity and PCOS are frequently associated with clinical manifestations such as hirsutism and menstrual irregularities. These manifestations are, to a higher extent in obese women, reversible by weight loss which leads to reduced levels of androgens and increased SHBG production [12]. Treatment strategies for both lean and obese women with PCOS include also lifestyle modifications such as the avoidance of pro-inflammatory agents, combined with pharmacological treatments such as combined oral contraceptives, anti-androgens, and insulin-sensitizing agents (e.g., metformin) [84].

#### 5.1.1. Pharmacological Therapies

Numerous meta-analyses have demonstrated the effectiveness of metformin in restoring ovulation [172,173], particularly in women who are resistant to clomiphene citrate which is used to block E2 negative feedback (an estimated 25% of women with PCOS [174]). Optimal dosage titration can help to minimize the adverse effects. However, due to heterogeneity in the metabolic profiles of women with PCOS, particularly in Phenotypes A and B, metformin is not recommended as a first-line infertility treatment. It remains beneficial for PCOS patients with coexisting metabolic syndrome and/or obesity [84]. Thiazolidinediones such as pioglitazone and rosiglitazone reduce plasma androgens and enhance insulin sensitivity, but their use is limited by the associated risks including increased coronary artery disease, myocardial infarction [84], and bladder cancer [175]. In animal models, metformin and pioglitazone improved ovarian function by modulating the AMP-activated protein kinase/PI3K/JNK pathway, promoting normal follicular development (with a higher percentage of early antral follicles) and reducing cystic follicles [176]. Combined treatment regimens, such as the aromatase inhibitor letrozole with metformin and pioglitazone, have demonstrated efficacy in women with clomiphene-resistant PCOS [177]. However, because letrozole suppresses E2 signaling, it is generally not recommended for ovulation induction in obese women, who often already exhibit low circulating E2 levels [12]. Letrozole may also block E2-mediated meiotic progression of the oocyte which would potentially limit its use in PCOS. Glucagon like peptide-1 (GLP-1) receptor agonists (GLP-1RAs), commonly prescribed for the treatment of type 2 diabetes mellitus, show promise for PCOS management due to their weight-reducing and anti-inflammatory properties [178]. In PCOS mouse models, GLP-1RAs have partially restored estrous cyclicity and improved MGC proliferation, partly through the inactivation of the pro-apoptotic FOXO1 [179].

#### 5.1.2. Nutritional Supplementation Therapies

As a complement to pharmacological approaches, nutritional supplementation therapies have been developed. Insulin resistance in obese women is correlated with vitamin D deficiency, probably linked to vitamin D sequestration in adipose tissue, which reduces its bioavailability [180]. Vitamin D, an essential modulator of oxidative stress and mitochondrial function, appears to attenuate chronic inflammation in PCOS by inhibiting the Nuclear factor kappa B (NF-κB) pathway [181,182]. Meta-analyses involving over 502 women reported that vitamin D supplementation increases dominant follicle number and menstrual regularity, particularly when combined with metformin [183]. In vitro studies further showed that vitamin D enhances FSH-induced MGC proliferation and steroidogenesis (by up-regulating the progesterone-producing enzyme) in animal and human models [184,185]. On the other hand, myo-inositol, a highly studied nutritional supplement, has demonstrated efficacy in improving insulin sensitivity and oocyte quality by facilitating glucose uptake through GLUT4 translocation and enhancing FSH-induced aromatase activity [186]. Meta-analyses have shown similar benefits as with metformin but with fewer side effects [186,187]. Spontaneous ovulatory rates were reported to increase significantly (25 to 65%) with myo-inositol, particularly in combination with folic acid treatment [186]. Co-administration with melatonin further improves oocyte quality and pregnancy outcomes [188], in accordance with enhanced implantation rates in PCOS oocytes matured in vitro with melatonin [189]. In that context, melatonin probably acts as a potential antioxidant to protect oocyte maturation [190].

### 5.2. Toward Safer and More Targeted Therapies

While current treatments such as metformin and anti-androgens are effective in managing hyperandrogenism and insulin resistance in women with PCOS, there is still a critical need for the development of more targeted therapeutic strategies. One promising approach involves the use of a neutralizing antibody targeting AMH receptor 2 (AMHR2). Preclinical studies have demonstrated that the administration of such an antibody in mice during mini-puberty can prevent the onset of PCOS-like features, while treatment in adults alleviates reproductive symptoms [191]. These findings highlight the potential of AMHR2-targeting therapy as both a preventive and a therapeutic option for PCOS. However, further investigations are necessary to assess the impact of such treatments on oocyte and blastocyst qualities, as well as their long-term safety and reproductive outcomes. Comprehensive clinical studies will be essential to determine whether this approach can be translated into a viable treatment for women with PCOS.

Emerging targets include factors activating SIRT3, such as the natural molecule sylibin, reported to display antioxidant and anti-inflammatory properties in animal models [192], or factors down-regulating the p21-activated kinase pathway [193], which may support mitochondrial function and reduce oxidative stress. Natural antioxidants such as vitamins C and E, selenium, polyphenols (e.g., quercetin, resveratrol), β-carotene, carnosol, and lycopene offer promising adjunctive therapies. These compounds help to reduce ROS levels in GCs, protecting oocyte maturation and enhancing IVF outcomes [138]. In rodent models, quercetin reduced insulin resistance by inhibiting NF-κB signaling in GCs [194], while resveratrol decreased AMH levels and increased ovarian glutathione content [195]. A 12-week combination therapy including inositols, antioxidants (lycopene and N-acetylcysteine), and vitamins demonstrated a significant improvement in menstrual regularity in both lean and obese PCOS patients [196]. However, large-scale clinical trials are necessary to confirm the efficacy and safety of these approaches across PCOS phenotypes that exhibit various metabolic features.

### 5.3. Improving Diagnosis and Personalization

Given the clinical heterogeneity of PCOS, phenotype-specific treatments should be set up. For instance, women with Phenotype D, characterized by milder metabolic and androgenic abnormalities [82], especially prevalent in certain populations such as Western India [197], may require less-intensive therapy. Diagnostic protocols should incorporate measurements of the levels of adipokines, vitamins, and calcium as well as oxidative markers in follicular fluid as potential biomarkers of oocyte quality and treatment response. As no current pharmacological therapy is free from long-term side effects, including risks of obesity, malignancy, and psychiatric disorders [198], there is an urgent need for new therapies that promote GC energy homeostasis and mitigate intracellular inflammation to increase the success of IVF outcomes in PCOS patients.

## 6. Conclusions

In summary, infertility in both obesity and PCOS in women is primarily associated with GC dysfunction. A key shared feature of these two conditions is insulin resistance, which is also present in lean women with PCOS, even those who do not exhibit hyperandrogenism. While hypogonadism in obese women results from low LH secretion, PCOS is characterized by elevated LH levels. These contrasting hormonal profiles, alongside the presence of insulin resistance in both conditions, support the hypothesis that insulin resistance may be a common underlying factor contributing to PCOS and obesity pathogenesis, regardless of androgen excess or BMI.

As outlined in Table 1, most studies investigating GC abnormalities have focused on overweight or obese women with PCOS, who represent the most prevalent cases. However, there is a lack of large-scale studies that simultaneously compare parameters from lean and obese women with PCOS and carefully analyze the phenotype of obese non-PCOS women. Such studies are essential to ensure standardized patient selection, consistent ovarian stimulation protocols, and reliable interpretation of the results. Indeed, translational research in this field remains heterogeneous, partly due to methodological inconsistencies. Human follicular samples are typically obtained after ovarian stimulation, which varies between individuals and clinical settings, introducing potential interpretation biases. Additional variability arises from differences in study design, patient selection, analytical techniques, and control for factors such as age, BMI, infertility etiology, and comorbidities. To advance our understanding and improve clinical outcomes, future studies must prioritize methodological standardization and detailed phenotypic stratification. These steps are critical for generating the robust, reproducible, and clinically relevant data that are required for the development of personalized therapeutic strategies for PCOS and obesity-related infertility. Additionally, given the ethnic and geographical variability in PCOS phenotypes and prevalence, conducting studies in diverse populations would be critical to uncover both shared and unique molecular mechanisms driving these disorders. Data from the substantial body of literature reviewed here has been summarized in a table (Table 1). The comparison of a large panel of clinical and molecular features of (lean and obese) PCOS and obese women notably suggests that the oocyte defects observed in women with PCOS, regardless of obesity status, may arise from metabolic alterations in GCs and associated changes in follicular fluid composition. Such defects compromise the ability of GCs to generate sufficient energy and antioxidants for proper oocyte maturation. Metabolic disturbances in GCs may also initiate downstream gene dysregulation in oocytes reported from the earliest stages of folliculogenesis [155,170]. Further work is needed to determine whether such metabolic alterations are also present in GCs from obese non-PCOS women. Whether GC impairments are secondary to systemic metabolic disturbances or not remains to be determined, however.

Emerging evidence highlights the role of the feto-maternal environment in the early programming of reproductive and metabolic disorders [199]. Maternal hyperandrogenism or metabolic imbalances during pregnancy may alter fetal development, predisposing offspring to insulin resistance and reproductive dysfunction later in life [200]. These hypotheses are increasingly supported by translational studies in animal models [191] and their study in humans should offer new insights to better our understanding and management of female reproductive health.

## Figures and Tables

**Table 1:** Comparison of metabolic defects and molecular dysregulations within GCs, and their impact on oocyte quality in obese women, as well as lean and obese women with PCOS, compared to controls (references are brackets). All references, except two, are derived from human studies.

Parameters	Obese Women	Obese PCOS Women	Lean PCOS Women
General Metabolic defects	Clinical insulin resistance [12,93]	Clinical and ovarian insulin resistance [82,84,85,107,143,145]	Ovarian insulin resistance associated or not with clinical insulin resistance [84,85,103,104,105,106,107,108,109,110,143,145]
	Systemic inflammation [12,93]	Systemic inflammation [86,103,112]	Systemic inflammation [86,103,112]
	Hyperandrogenism (not a majority) [98]	Hyperandrogenism (depending on PCOS phenotype) [81,82]	Hyperandrogenism (depending on PCOS phenotype) [81,82]
	Hyperlipidemia [12,93]	Hyperlipidemia [8,111]	Hyperlipidemia [8,102,111]
GC metabolic dysfunction	N/D	↑ Insulin receptor [85,148]	↓ or unchanged insulin receptor [85,92,144]
	N/D	↓ Insulin-mediated metabolic signaling [143,145]	↓ Insulin-mediated metabolic signaling [143,145]
	N/D	↑ Inflammatory-related genes [146,147,150]	↑ Inflammatory-related genes [150,151]
	N/D	↓ Glycolysis-related genes [5,78]	↓ Glycolysis-related genes [6,136]
	↓ Mitochondrial respiration [108]	N/D	↓ Mitochondrial respiration [6,136]
	N/D	N/D	↓ Protein acetylation [140]
Gonadotropin receptors	↓ FSHR expression [126]	↓ FSHR expression [126]	N/D
	N/D	↑ LH sensitivity [92]	↑ LH sensitivity [92]
Altered oocyte-GC communication 1/Structural changes 2/Follicular fluid abnormalilies 2/Follicular fluid abnormalilies (continues)	N/D	↓ Aquaporin 9 [153]	↓ Aquaporin 9 [153]
	N/D	N/D	↓ Gap junction [155]
	N/D	ECM dysregulation [147,156]	N/D
	↑ FFAs and cytokines [12,15,117]	↑ FFAs and cytokines [146,150]	↑ FFAs and cytokines [151]
	N/D	↓ Pyruvate and lactate [5,78]	↓ Pyruvate and lactate [6,143]
	↑ Leptin and adipokines [12,117,118]	↑ Leptin and adipokines [169]	↑ Leptin and adipokines [169]
	↑ C-reactive protein [17]	↑ C-reactive protein [146]	N/D
	N/D	↓ Carnitine [141]	↓ Carnitine [141]
	N/D	↓ Melatonin [158]	↓ Melatonin [158]
	N/D	↑ AMH [162]	↑ AMH [162]
	N/D	↑ T [158,160,161]	↑ T [157,158]
	N/D	↓ E2 [126,158,159]	unchanged E2 [157]
	N/D	↓ P4 [157,158]	↓ P4 [160,161]
GC oxidative stress and apoptosis	↑ Apoptotic markers (mouse model) [127]	↑ Apoptotic markers [5,130,132,137]	↑ Apoptotic markers [130,131,133]
	N/D	↑ Inflammatory genes [147,150]	↑ Inflammatory genes [150]
	↓ ATP and ROS [117]	↓ ATP and ROS [5,78,137]	↓ ATP and ROS [6,78,133,135,136,151]
	↓ Mitochondrial integrity [42]	↓ Mitochondrial integrity [5,135]	↓ Mitochondrial integrity [136]
	N/D	↓Oxidative stress response genes [156]	↓ Oxidative stress response genes [6,155]
	Unchanged mtDNA (mouse model) [125]	↓ mtDNA [135]	↓ mtDNA [78,135,136]
	N/D	N/D	↓ Signaling inducing proliferation [48,155]
Impaired oocyte quality	↓ Competence [13,17]	↓ Competence [5,78,137]	↓ Competence [136,137]
	Spindle defects, aberrant chromosome segregation [124]	Altered meiotic gene expression [170]	Altered meiotic gene expression [170]

FFAs, free fatty acids; ECM, extracellular matrix; LH, luteinizing hormone; FSH, follicle-stimulating hormone; mtDNA, mitochondrial DNA; N/D, not determined. ↑: increases. ↓: decreases.

## Data Availability

Not applicable.

## Abbreviations

AMH: anti-Müllerian hormone; AR: androgen receptor; ATP: adenosine triphosphate; BMP15: bone morphogenetic protein-15; BMI: body mass index; Ca2+: calcium ion; COC: cumulus–oocyte complex; CGCs: cumulus granulosa cells; cAMP: cyclic adenosine monophosphate; cGMP: cyclic guanosine monophosphate; E2: 17β-estradiol; EGF: epidermal growth factor; ECM: extracellular matrix; ER: estrogen receptor; ERK: extracellular signal-regulated kinase; FSH: follicle-stimulating hormone; GCs: granulosa cells; GDF9: growth differentiation factor-9; IGF-I: insulin-growth factor; IVF: in vitro fertilization; LH: luteinizing hormone; MAPK: mitogen-activated protein kinase; MGCs: mural granulosa cells; MPF: maturation-promoting factor; mtDNA: mitochondrial DNA; NPPC: natriuretic peptide precursor; NPR2: NPPC receptor; PDE3A: phosphodiesterase 3A; PI3K: phosphoinositide-3 kinase; pHi: intracellular pH; P4: progesterone; PR: progesterone receptor; ROS: reactive oxygen species; scRNA-seq: single-cell RNA sequencing; T: testosterone; TGFβ: transforming growth factor beta; TZP: transzonal cytoplasmic process.
